# Prevention of Device-Related Healthcare-Associated Infections

**DOI:** 10.12688/f1000research.7493.1

**Published:** 2016-01-14

**Authors:** Edward J. Septimus, Julia Moody

**Affiliations:** 1 Texas A&M Health Science Center, Houston, Texas, 77005, USA; 2 Clinical Services Group, Hospital Corporation of America, Nashville, Tennessee, 37203, USA

**Keywords:** CLABSI, central line–associated bloodstream infections, healthcare-associated infections, CAUTI, catheter-associated urinary tract infections

## Abstract

Healthcare-associated infections (HAIs) are a leading cause of morbidity and mortality in hospitalized patients. Up to 15% of patients develop an infection while hospitalized in the United States, which accounts for approximately 1.7 million HAIs, 99,000 deaths annually and over 10 billion dollars in costs per year. A significant percentage of HAIs are preventable using evidenced-based strategies. In terms of device-related HAIs it is estimated that 65-70% of catheter-line associated bloodstream infections (CLABSIs) and catheter-associated urinary tract infections (CAUTIs) are preventable. To prevent CLABSIs a bundle which includes hand hygiene prior to insertion and catheter manipulation, use of chlorhexidene alcohol for site preparation and maintenance, use of maximum barrier for catheter insertion, site selection, removing nonessential lines, disinfect catheter hubs before assessing line, and dressing changes are essential elements of basic practices. To prevent CAUTIs a bundle that includes hand hygiene for insertion and catheter or bag manipulation, inserting catheters for appropriate indications, insert using aseptic technique, remove catheters when no longer needed, maintain a close system keeping bag and tubing below the bladder are the key components of basic practices.

## Introduction

Health care-associated infections (HAIs) are a leading cause of morbidity and mortality in hospitalized patients. Up to 15% of patients develop an infection while hospitalized. In the US, this accounts for approximately 1.7 million HAIs and 99,000 deaths annually. HAIs are now the fifth leading cause of death in US acute care hospitals ^1^. A recent report estimated US health care system costs attributable to the five most common HAIs—central line-associated bloodstream infections (CLABSIs), catheter-associated urinary tract infections (CAUTIs), ventilator-associated pneumonia, surgical site infection, and *Clostridium difficile* infection—to be $9.8 billion, even without considering the sizable societal costs ^2^. We now know a significant percentage of HAIs are preventable by using evidence-based strategies ^3^. In terms of device-related HAIs, it is estimated that 65% to 70% of CLABSIs and CAUTIs are now preventable. There is now coordination of federal efforts aimed at HAI prevention, including public reporting of hospital-specific HAI rates and linking hospital-specific HAI performance to financial reimbursement as a strategy to motivate hospitals’ HAI prevention efforts. Since 2011, hospitals have been required to report CLABSIs among patients in intensive care units (ICUs) to the Centers for Disease Control and Prevention’s (CDC) National Healthcare Safety Network (NHSN) in order to qualify for annual payment updates. CAUTI reporting in ICUs was added in 2012. In addition, starting in 2015, CLABSIs and CAUTIs are reported in general medical surgical wards. Along with other quality metrics, these HAI data will be used to determine hospital-specific Centers for Medicare & Medicaid Services reimbursement levels as part of value-based purchasing, thereby shifting reimbursement from volume-driven to performance-driven.

Evidence-based recommendations provided through guidelines or guidance documents form the foundation for HAI prevention efforts. Translating knowledge into practice requires an integrated approach to address both technical and adaptive work, including a deep understanding of the health care delivery system and human behavior, fostering engagement and ownership of the improvement process by local interdisciplinary teams, creating centralized support for the technical work, encouraging local adaptation of the intervention bundle, and ensuring a collaborative culture within the facility ^4^. The use of prevention bundles has been shown to reduce HAI rates. A bundle is best defined as a grouping of evidence-based practices that individually improve care. A number of studies have demonstrated the impact of catheter insertion and maintenance bundles on CLABSI rates and have shown that CLABSI prevention bundles are effective, sustainable, and cost-effective for both adults and children ^5, 6^. Bundles have also been used in successful multifaceted efforts to reduce CAUTI ^7^. The purpose of this article is to review the strategies to prevent device-related infections, CLABSIs and CAUTIs.

## Catheter line-associated bloodstream infection

An estimated 41,000 CLABSIs occurred in the US in 2009 ^8^. Although the primary focus over the last two decades has been the ICU, the majority of CLABSIs occur outside the ICU ^9^. In 2013, Zimlichman *et al.* published a meta-analysis of costs and financial impact of HAIs on the US health care system ^2^. On a per-case basis, CLABSIs were found to be the most costly HAIs at $45,814.

There are two major sources for contaminated catheters and subsequent CLABSI: (1) colonization at the insertion site with migration of organism(s) along the external surface of the catheter is the most common source of CLABSI in catheters within the first week of catheterization, and (2) direct contamination of connectors/hubs resulting in internal colonization and subsequent CLABSI is the major source in catheters in place for at least 1 week. Less commonly, catheters can be seeded hematogenously from another site of infection and rarely from contaminated intravenous (IV) fluids ^10^.

CLABSI is a term used for surveillance purposes by the CDC’s NHSN to identify bloodstream infections (BSIs) that occur in patients with a central venous line. A central line is an intravascular catheter that terminates at or close to the heart or in one of the great vessels which is used for infusion, withdrawal of blood, or hemodynamic monitoring. A laboratory-confirmed CLABSI (LCBI) is where a central line was in place for more than 2 calendar days on the date of event, with day of device placement being day 1, and a central line was in place on the date of event or the day before. If a central line was in place for more than 2 calendar days and then removed, the date of event of the LCBI must be the day of discontinuation or the next day. There are two criteria: (1) the patient has a recognized pathogen cultured from one or more blood cultures, and the organism cultured from blood is not related to an infection at another site, or (2) the patient has at least one of the following signs or symptoms: fever (>38.0°C), chills, or hypotension and the organism cultured from blood is not related to an infection at another site, and the same common commensal—i.e. diphtheroids ( *Corynebacterium* spp. not *C. diphtheriae*), *Bacillus* spp. (not *B. anthracis*), *Propionibacterium* spp., coagulase-negative staphylococci (including *S. epidermidis*), viridans group streptococci, *Aerococcus* spp., and *Micrococcus* spp.—is cultured from two or more blood cultures drawn on separate occasions ^11^.

Prevention strategies are divided into insertion and maintenance, as well as basic practices and special approaches. Basic practices should be implemented in all acute care hospitals, whereas special approaches should be considered only when CLABSIs are not controlled by use of basic practices ^12^ ( Table 1).

**Table 1.** Strategies to prevent catheter line-associated bloodstream infections.

Insertion 1. Hand hygiene 2. Chlorhexidine gluconate (CHG)-alcohol for site preparation 3. Maximum sterile barrier precautions during central-line insertion 4. Site selection 5. Ultrasound guidance
Maintenance 6. Disinfect catheter hub before accessing the catheter. 7. Promptly remove catheters when no longer needed. 8. Bathe intensive care unit patients over 2 months of age with CHG on a daily basis. 9. Change transparent dressing and perform site care with a CHG-based product every 5 to 7 days, or every 2 days for a gauze dressing. 10. Replace dressing immediately if dressing becomes damp, loose, or visibly soiled. 11. Replace administration sets not used for blood, blood products, total parenteral nutrition (TPN), or lipids at intervals of no longer than 96 hours. Administration sets used for blood, blood products, TPN, or lipids should be changed every 24 hours.
Special approaches 12. Use a chlorhexidine/silver sulfadiazine- or minocycline/rifampin-impregnated central line. 13. Use a CHG-containing dressing for central venous catheters in patients over 2 months of age. 14. Use an antiseptic-containing cap to cover connectors. 15. Antimicrobial lock solutions in select populations.

### Insertion

1. Have a process, such as a checklist, to ensure compliance with evidence-based infection prevention strategies to prevent CLABSIs.
2. Perform hand hygiene before and after insertion, dressing change, or hub access. Multiple studies have documented reduction of HAIs, including CLABSIs, with strict adherence to hand hygiene. A recent publication reported a decrease in CLABSI rates from 4.08 per 1000 catheter line days to 0.42 per 1000 catheter line days after a multifactorial action plan to improve hand hygiene compliance ^13^.
3. Use a chlorhexidine gluconate (CHG)-alcohol antiseptic for skin preparation. Multiple studies show a greater reduction in both colonization and infection by using CHG-alcohol compared with a povidone-iodine (PI) preparation ^14^. A recent study compared CHG-alcohol with PI-alcohol for prevention of CLABSIs. CHG-alcohol was associated with a significantly lower incidence of CLABSI compared with PI-alcohol (0.28 versus 1.77 per 1000 catheter days; hazard ratio 0.15, 95% confidence interval [CI] 0.05 to 0.41; *P* = 0.0002) ^15^.
4. Use maximum sterile barrier (MSB) precautions during central line insertion. MSB precautions include mask, cap, sterile gown, and sterile gloves by all health care workers involved in the catheter insertion. In addition, the patient should be covered with a sterile full-body drape during insertion. Most studies have shown a reduction in CLABSIs when maximum barrier precautions are enforced. In a randomized control trial, MSB precautions during insertion of central venous catheter (CVC) were compared with sterile gloves and a small drape. The MSB group had fewer episodes of both catheter colonization (relative risk [RR] = 0.32, 95% CI 0.10 to 0.96, *P* = 0.04) and CLABSIs (RR = 0.16, 95% CI 0.02 to 1.30, *P* = 0.06) ^16^. However, a recent prospective randomized trial in surgical patients failed to show a benefit of maximum barrier precautions ^17^. Nonetheless, the majority of evidence suggests a reduction in CLABSI rates with maximum barrier precautions.
5. Avoid using the femoral site for central venous pressure (CVP) access in adult obese patients when placed under elective and controlled situations ^12, 18^. In pediatrics, the risk of infection is equal in femoral site insertion versus non-femoral sites ^19^.
6. Use ultrasound guidance for internal jugular catheter insertion. Ultrasound use has been shown to reduce the risk of CLABSI and non-infectious complications during insertion ^20^. In a recent systemic review and meta-analysis, ultrasound-guided subclavian insertion also reduced the risk of adverse events ^21^.

### Maintenance

1. Disinfect catheter hubs and connectors before accessing the catheter. Recent studies suggest that friction for at least 5 seconds is needed for reducing contamination of split-septum needless connectors ^22^. Monitoring scrubbing of the hub is important to ensure compliance.
2. Promptly remove catheters when no longer needed, since prolonged catheter use increases the risk of CLABSI ^23^. Multiple concurrent lines are also associated with increased risk for CLABSIs ^24^. In addition, central lines placed under conditions that are not compliant with asepsis (emergently placed) should be replaced or removed whenever feasible. Therefore, facilities should monitor all lines for indications on a daily basis.
3. Minimize unnecessary manipulation of lines, such as drawing blood through lines for convenience.
4. Bathe ICU patients over 2 months of age with CHG on a daily basis. In the last few years, there has been growing evidence that daily CHG bathing in the ICU reduces CLABSIs in both adult ICUs and pediatric ICUs. Milstone *et al.* found that 2% CHG cloth bathing was significantly associated with a significant decline in BSIs compared with standard bathing ^25^. In another trial, called the REDUCE MRSA (Randomized Evaluation of Decolonization versus Universal Clearance to Eliminate MRSA), universal decolonization with daily CHG with 2% CHG cloths along with 5 days of mupirocin was compared with targeted decolonization or screening and isolation alone. The trial demonstrated universal decolonization was more effective than targeted decolonization or screening and isolation in significantly reducing all cause bloodstream infection by 44% (P<0.001) ^26^. The role of CHG bathing outside the ICU remains to be determined.
5. Change transparent dressing and perform site care with a CHG-based product every 5 to 7 days or every 2 days for a gauze dressing ^12^. Replace dressing immediately if dressing becomes damp, loose, or visibly soiled. Recently, Timsit *et al.* found that the number of dressing disruptions was related to increased risk of colonization around the insertion site and that the risk of CLABSI increased threefold after the second dressing disruption ^27^.
6. Replace administration sets not used for blood, blood products, total parenteral nutrition (TPN), or lipids at intervals of no longer than 96 hours. Administration sets used for blood, blood products, TPN, or lipids should be changed every 24 hours ^12^.

### Special approaches

1. Antimicrobial-/antiseptic-impregnated catheters: Use of a chlorhexidine/silver sulfadiazine- or minocycline/rifampin-impregnated CVC in adults whose catheter is expected to remain in place for more than 5 days has been shown to reduce the risk of CLABSIs ^28, 29^.
2. Use a CHG-containing dressing for CVPs in patients over 2 months of age: in a large multicenter randomized controlled trial, the investigators compared chlorhexidine-impregnated sponge dressing versus standard dressings in ICU patients. They found a significant reduction in CLABSIs (6/1953 catheters, 0.40 versus 17/1825 catheters, 1.3 per 1000 catheter-days; hazard ratio 0.24, 95% CI 0.09 to 0.65) ^30^. In a follow-up study, the authors published a large randomized trial using a CHG-gel-impregnated transparent dressing and also found a significant decrease in CLABSIs ^31^.
3. Use an antiseptic-containing cap to cover connectors: in an observational trial in a tertiary care unit, the practice of central line hub care was changed from cleaning with alcohol wipes to using alcohol-impregnated protectors. The implementation of alcohol-impregnated protectors significantly reduced the rate of CLABSIs. The rate of CLABSIs decreased from 2.3/1000 central line-days in the preintervention period to 0.3/1000 central line-days in the intervention period (RR 0.14; 95% CI 0.02 to 1.07; *P* = 0.03) ^32^. Wright *et al.* published a multifacility, quasi-experimental study of adult patients with central lines divided into P1 (baseline), when the standard scrub was used; P2, when an alcohol cap was used on all central lines; and P3, when standard disinfection was reinstituted. CLABSI rates declined from 1.43 per 1000 line-days (16/11,154) to 0.69 (13/18,972) in P2 ( *P* = 0.04), and increased to 1.31 (7/5354) in P3 ^33^. Some of the studies had blood cultures drawn from lines; therefore, some reduction may have been reduction of colonization rather than true infection.
4. Antimicrobial lock solutions: to use lock solutions, supratherapeutic concentrations of an antimicrobial solution are allowed to dwell rather than simply flush through the catheter. Owing to concerns regarding potential for emergence of resistance, antimicrobial locks have been reserved for patients with long-term hemodialysis catheters, patients with limited IV access and a history of recurrent CLABSIs, and patients at risk for severe sequelae, such as those with recently implanted intravascular devices (e.g. prosthetic heart valve or intravascular graft) ^12^. In a recent meta-analysis, Zacharioudakis *et al.* reported on 23 studies involving adult patients undergoing hemodialysis, adult and pediatric oncology patients, neonates, and patients receiving parenteral nutrition ^34^. The authors found that the use of antimicrobial lock solutions led to a 69% reduction in CLABSI rate (RR 0.31, 95% CI 0.24 to 0.40) and a 32% reduction in the rate of exit site infections (RR 0.68, 95% CI 0.49 to 0.95) compared with heparin. They concluded that antimicrobial lock solutions are effective at reducing CLABSIs in select populations and are additive to basic practices ^34^. Owing to concern over the development of antimicrobial resistance, there has been recent interest in non-antibiotic antimicrobial solutions such as ethanol ^35^. The effectiveness of ethanol has been studied almost exclusively in hemodialysis patients, demonstrating a reduction of CLABSIs with long dwell times.

## Catheter-associated urinary tract infection

Urinary tract infections (UTIs) are common hospital-acquired infections accounting for 15% of HAIs, and approximately 70% are associated with an indwelling urethral catheter. Up to 16% of inpatients have a urinary catheter at some point during their admission ^1^. Although the primary focus over the last two decades has been the ICU, the majority of CAUTIs occur outside the ICU ^36^. In 2013, Zimlichman *et al.* published a meta-analysis of costs and financial impact of HAIs on the US health care system ^2^. On a per-case basis, CAUTIs cost at least at $896. The incidence rate was third highest among US adult inpatients. In addition, there are many non-infectious complications that are at least as common as urinary tract infections (UTIs). Urinary catheters can operate as physical restraints by reducing mobility, leading to increased falls, risk of venous thromboembolism, and pressure ulcers ^37^. Leakage, urethral strictures, gross hematuria, and blockage have also been reported ^38^.

The major contributing risk for developing CAUTI is the duration the urinary device is present. Other risk factors include meatal colonization with uropathogens, microbiological colonization of the draining bag, and gaps in catheter insertion and care. 

CAUTI is a term used for surveillance purposes by the CDC’s NHSN to identify UTIs that occur in patients with an indwelling Foley urinary catheter. A laboratory-confirmed CAUTI is where the indwelling urinary catheter is in place for more than 2 calendar days on the date of event, with day of device placement being day 1, and an indwelling urinary catheter was in place on the date of event or the day before. If an indwelling urinary catheter was in place for more than 2 calendar days and then removed, the date of event of the CAUTI must be the day of discontinuation or the next day. In 2015, two significant changes occurred in the surveillance definitions: (1) retirement of cultures with less than 100,000 colony-forming units (CFU)/ml and positive urinalysis diagnostic tests, and (2) removal of Candida species and yeast in the qualifying urinary pathogen list.

The 2015 surveillance criteria for symptomatic UTI in adults are the patient has a recognized pathogen cultured from urine of no more than two organism species, where at least one organism is a bacteria of more than 100,000 CFU/ml and the patient has at least one of the following signs or symptoms: fever (>38.0°C), suprapubic tenderness, costovertebral angle pain or tenderness, urinary urgency, urinary frequency or dysuria, where symptoms except for fever have no other recognized cause within the infection window period ^39^. Prevention strategies are divided into basic practices of insertion, maintenance, and removal and should be implemented in all acute care hospitals ^40^ ( Table 2).

**Table 2.** Strategies to prevent catheter-associated urinary tract infections.

Insertion • Assess for medical necessity and appropriateness of device. • Hand hygiene • Cleanse or perform pericare to remove gross material prior to applying antiseptic solution. • Use aseptic insertion technique and sterile supplies. • Apply securement device to prevent movement and traction.
Maintenance • Hand hygiene before and after catheter or bag manipulation. • Regular pericare and incontinence care to keep catheter clean. • Maintain a sterile, continuous closed system. • Maintain unobstructed flow, minimizing dependent loops and kinks. • Keep bag below bladder, including during transport. • Collect urine from the port, not drain tubing. • Empty drain bag regularly by using a patient-dedicated collection container. • Assess daily for medical necessity and appropriateness of device. • Replace devices when breaks or leaks occur with the catheter and collection system.
Removal • Remove when no longer medically necessary. • Implement approved device removal protocols (intensive care unit, wards, and post-anesthesia care unit). • Support toileting and consider alternative urinary devices. • Use bladder scanners to assess urinary retention.

### Insertion

1. Establish processes to assess for medical necessity for patient care and appropriateness of device in care areas such as emergency room, ICU, non-ICU ward, perioperative suites, and inserting urinary catheters. Table 3 includes suggested indication for urinary catheter insertion ^40^. Consider alternative devices or intermittent catheterization when applicable. One statewide effort reduced catheter device utilization through education and monitoring compliance with appropriate indications ^41^.
2. Perform hand hygiene before and after insertion.
3. Cleanse or perform pericare to remove gross material prior to applying antiseptic solution to maximize antiseptic bioactivity and minimize introduction of bacteria, fluids, and secretions into the bladder.
4. Use aseptic insertion technique and sterile supplies. All-inclusive prepackaged kits or having necessary supplies conveniently located support aseptic insertion and evidence-based practice.
5. Apply securement device to prevent movement and traction.

**Table 3.** Appropriate indications for insertion of a urinary catheter.

• Perioperative use for selected surgical procedures, such as urologic surgery or surgery on contiguous structures of the genitourinary tract; prolonged surgery; large-volume infusions or diuretics during surgery; or intraoperative monitoring of urine output needed. • Hourly assessment of urine output in patients in an intensive care unit. • Management of acute urinary retention and urinary obstruction. • Assistance in healing of open pressure ulcers or skin grafts for selected patients with urinary incontinence. • As an exception, at patient request to improve comfort (e.g. end-of-life care)

### Maintenance

1. Perform hand hygiene before and after catheter or bag manipulation.
2. Perform regular pericare and incontinence care to keep catheter clean.
3. Practice bundles to optimize unobstructed urine flow include the following:
  ○ Maintaining a sterile, continuous closed system.
  ○ Minimizing dependent loops and kinks.
  ○ Keeping bag below bladder, including during transport.
  ○ Emptying urine from the collecting bag regularly by using a patient-dedicated collection container.
4. Collect urine samples from the port, not drain tubing.
5. Replace devices when breaks or leaks occur with the catheter and collection system.
6. Assess daily for medical necessity for patient care. Consider other methods such as intermittent catheterization where appropriate.

### Removal

1. Remove when no longer medically necessary. Reminders, stop orders, and protocolizing removal for patients meeting appropriate indications have been shown to be successful in timely removal of urinary catheters when no longer necessary for patient care ^42, 43^. Successful outcomes have occurred when partnering with clinicians and nurses to adopt removal protocols in qualifying patients upon change in level of care from ICU to non-ICU wards, or post-anesthesia care unit to inpatient units. Directives that allow nurses to remove urinary catheters on the basis of specified criteria have been shown to reduce catheter days and CAUTIs ^44^.
2. Support toileting and consider alternative urinary devices such as intermittent catheterization, external male condom catheters, and urinals.
3. Use bladder scanners to assess urinary retention.

### Approaches excluded from routine catheter-associated urinary tract infection prevention

1. Use of antimicrobial-/antiseptic-impregnated catheters is unproven for routine use. Pickard *et al.* published a prospective randomized three-arm trial comparing a standard latex catheter, latex silver alloy-coated catheter and a nitrofurazone silicone-impregnated catheter ^45^. They found no differences in symptomatic culture-confirmed urinary infection at 6 weeks with use of the two latex catheters and only a small decrease with the nitrofurazone silicone catheter (odds ratio 0.68, 97.5% CI 0.48 to 0.99; *P* = 0.017). However, the nitrofurazone catheter was associated with greater patient discomfort and increased catheter removal. They concluded that routine use of antimicrobial-impregnated catheters was not supported ^45^.
2. Avoid screening for asymptomatic bacteriuria (ABU) in catheterized patients. Studies have confirmed that overtreatment of ABU with urinary catheters remains high and can lead to increased antimicrobial resistance and *C. difficile* infections. Recently, Trautner *et al.* demonstrated that an intervention to decrease inappropriate screening for ABU significantly decreased overtreatment of ABU ^46^.
3. Do not change catheters routinely.

## Summary

Effective sustainable reduction of CLABSIs and CAUTIs requires a multifaceted approach implementing evidence-based strategies plus an adaptive approach which aligns health care professional behaviors. We have made significant progress in the last 10 years, but more can be done in eliminating preventable CLABSIs and CAUTIs.
